# Visual Outcomes Three and Six Months After Bilateral Implantation of Extended Monofocal Isopure 1.2.3 Lenses

**DOI:** 10.3390/jcm15145506

**Published:** 2026-07-14

**Authors:** Wojciech Lubiński, Maria Strojny, Karolina Podborączyńska-Jodko, Urszula Danes-Bogacka, Maciej Mularczyk

**Affiliations:** 1 II Department of Ophthalmology, Pomeranian Medical University, 70-111 Szczecin, Poland; 2 Chair and Department of Human and Clinical Anatomy, Pomeranian Medical University, 70-111 Szczecin, Poland

**Keywords:** extended depth-of-focus, Isopure 1.2.3 IOL implantation, visual outcomes comparison, neuroadaptation

## Abstract

**Objectives**: The objective of this study was to compare visual outcomes three and six months after bilateral implantation of extended monofocal Isopure 1.2.3 lenses. **Methods**: Prospective study: 20 patients (40 eyes) aged 51–75 years underwent uncomplicated bilateral cataract surgery with the implantation of Isopure 1.2.3 lenses. Three and six months after surgery, the following examinations were performed: monocular and binocular UDVA, UIVA, UNVA, contrast sensitivity, patient satisfaction, spectacle independence, incidence of photic phenomena, and defocus curve (only 6-month assessment). **Results**: Three months after surgery, the means of monocular and binocular UDVA were 0.06 ± 0.08 logMAR and 0.02 ± 0.07 logMAR, respectively. The mean binocular UIVA values were 0.15 ± 0.12 logMAR (66 cm) and 0.19 ± 0.11 logMAR (80 cm), and those for UNVA 0.32 ± 0.12 logMAR (40 cm). Binocular contrast sensitivity was within the normal range. Defocus curve indicated prolonged depth of focus for intermediate vision. High level of patient satisfaction, low frequency and intensity of photic phenomena, and significant independence from glasses were achieved. Six months postoperatively, the visual outcomes and patient satisfaction did not differ significantly from the three-month follow-up. **Conclusions**: Bilateral implantation of Isopure 1.2.3 IOLs provides very good distance, good intermediate and acceptable near vision. With low incidence and perception level of photic phenomena, as well as significant spectacle independence, it should be considered an option for patients not suitable for multifocal IOLs. The investigated visual outcomes are stable starting from the third postoperative month, which could be the result of good neuroadaptation in a short period of time.

## 1. Introduction

Cataract is a very common disease affecting an estimated 95 million people worldwide [1]. Most patients undergoing cataract surgery receive a monofocal intraocular lens (IOL) and are spectacle-dependent. Some patients receive multifocal IOLs, reducing dependence on glasses, but they should accept decreased optical performance under low-light conditions, increased dysphotopsias and reduced overall quality of vision [2,3]. Enhanced monofocal lenses (monofocal-plus) represent a solution to these limitations, providing functional vision over a broad range of distances with few photic phenomena. On visual inspection, the monofocal-plus IOLs are indistinguishable from monofocal IOLs and are characterized by a unique aspheric posterior or anterior surface [4,5]. Enhanced monofocal IOLs are designed to provide excellent uncorrected distance visual acuity (UDVA) while maintaining good uncorrected intermediate visual acuity (UIVA) and acceptable near visual acuity without the visual quality disturbances associated with multifocal IOLs [2,3]. An additional advantage over multifocal IOLs might be the shorter period of neuroadaptation to new optical conditions, which last at least 6 months for multifocal IOLs [6,7]. A good example of monofocal-plus IOL is Isopure 1.2.3 IOL, a refractive, aspherical, monofocal based on isofocal technology.

The goal of this study was to evaluate the visual outcomes and assess the neuroadaptation time in patients after bilateral implantation of Isopure 1.2.3 IOLs by analysis of the results 3 and 6 months after the surgery.

## 2. Patients and Methods

### 2.1. Study Design

This prospective descriptive study, with follow-up assessments conducted at 3 and 6 months, was carried out at the II Department of Ophthalmology, Pomeranian Medical University (PMU), Szczecin, Poland. This study was performed in accordance with the Declaration of Helsinki and was approved by the local Ethics Committee of PMU (KB-006/07/2023-A-16). All patients provided written informed consent.

### 2.2. Study Population

Forty eyes of 20 patients with a mean age of 66.7 years underwent successful uncomplicated cataract surgery with implantation of monofocal-plus Isopure 1.2.3 lenses.

Inclusion criteria were as follows: bilateral cataract, absence of additional ocular and systemic diseases with a known influence on visual system function, presbyopia/hyperopia, pupil size of 3–6 mm in dim light, preoperative corneal astigmatism lower than 0.75 D, and strong motivation for spectacle independence while accepting the possible need for slight spectacle correction for near vision. Exclusion criteria included: patients under 50 or over 75 years, impaired ocular motility, unrealistic visual outcome expectations, professions requiring high visual precision (e.g., watchmakers), psychiatric disorders, history of stroke, dissatisfaction with progressive spectacles, and the need for IOL beyond the available diopter range (+10.0 to +35.0 D).

### 2.3. IOLs and Surgical Technique

In this study, the implanted IOL was the Isopure 1.2.3 (BVI, PhysIOL S.A., Liege, Belgium)—an EDOF lens, a fully refractive, aspheric, preloaded lens with four closed-loop, hydrophobic acrylic glistening-free materials. The unique polynomial technology used in this lens provides improved intermediate vision without inducing severe photic phenomena [8].

Target refraction was emmetropia/−0.25 D and IOL power calculations were performed using IOL Master (Carl Zeiss—Meditec, Jena, Germany: software version 2025) with Barret Universal II formula. The same experienced surgeon performed under topical anesthesia a bimanual Microincision Cataract Surgery (Ngenuity 3D) with two clear corneal incisions (at the 2 and 10 o’clock positions) of 1.2 × 1.4 mm, an anterior capsulorhexis diameter of approximately 5 mm, an ultrasound phacoemulsification (Stellaris –Bausch & Lomb). The Isopure 1.2.3 was implanted using a pressure-release system through temporal incision, which was enlarged to 2.2–2.3 mm. The fellow eye was operated 1 month after the first one. For three weeks after surgery, topical moxifloxacin, bromfenac, dexamethasone and lubricants were administered.

### 2.4. Outcome Measures

Before surgery and at 3 and 6 months postoperatively, we evaluated patients for the following measures: biomicroscopic examination of anterior and posterior segments of the eye, intraocular pressure, postoperative complications, binocular and monocular uncorrected and distance-corrected visual acuity in logMAR units (UDVA, DBCVA) from 4 m, binocular and monocular uncorrected and corrected intermediate visual acuity (UIVA, BCIVA—66, 80 cm), and binocular and monocular uncorrected and the best-corrected near visual acuity in logMAR units (UNVA, BCNVA). Three and six months after the surgery we additionally assessed spectacle independence, binocular photopic (85 cd/m^2^), mesopic (3 cd/m^2^) distance (2.5 m) and binocular photopic (85 cd/m^2^) near (40 cm) uncorrected contrast sensitivity (CS;1.5,3,6,12,18 c/deg), quality of vision using a modified Visual Function Questionnaire (VFQ-25), and the frequency and perceived severity of glare and halo. The defocus curve was evaluated only 6 months after surgery.

### 2.5. Statistical Analysis

The assumption of normal distribution was checked on the differences in values between the two time points using the Shapiro–Wilk W test. Descriptive statistics were presented as mean, SD, minimum, and maximum values. Differences between the two time points were compared using the Wilcoxon signed-rank test. The right and left eyes were analyzed separately, and no pooled statistical analyses including all 40 eyes as independent observations were performed. The significance level was set at *p* < 0.05.

## 3. Results

### 3.1. Patients’ Demographic Data and Preoperative Biometric Characteristic

Patients’ age ranged from 51 to 75 years, with a mean age of 66.7 years. Fourteen participants were female (70%) and six participants were male (30%). A summary of the patients’ demographic and preoperative biometric characteristics is presented in Table 1.

Before surgery and during the postoperative follow-up visits, both the anterior and posterior ocular segments were examined. Apart from cataract (LOCS III, NO2, range 2.5–3), no abnormalities were observed before and postoperatively in the anterior segment of the eye. The fundus was normal in all analyzed patients. There were no complications after the implantation. The intraocular pressure remained within the normal range in all patients—mean before the surgery: 16.54 ± 2.08 mmHg; after 3 months: 15.80 ± 2.32 mmHg; after 6 months: 14.53 ± 1.94 mmHg. In all patients, the lens centration was correct, and there were no signs of posterior capsule opacification in the time of follow-up requiring Nd:YAG posterior capsulotomy.

### 3.2. Visual Acuity Outcomes

Three months after surgery, the mean binocular UDVA was 0.02 ± 0.07 logMAR, while the mean binocular DBCVA was −0.02 ± 0.06. The mean binocular UIVA at 66 and 80 cm was 0.15 ± 0.12 logMAR and 0.19 ± 0.11 logMAR, respectively, and the mean binocular UNVA was 0.32 ± 0.12 logMAR. Table 2 presents the monocular and binocular visual acuity results obtained three and six months after surgery. No statistically significant differences were found between the two follow-up points. Table 3 presents the results of binocular and monocular DBCVA, BCIVA and BCNVA three and six months after surgery. Statistical differences between two follow-up points were not found.

### 3.3. Refractive Outcomes

Preoperatively, the mean refractive spherical equivalent (MRSE) was −0.91 ± 2.63 D (range: −5.25 to +3.25 D) for the right eye and −0.23 ± 2.31 D (range: −4.25 to +2.75 D) for the left eye. Three months after surgery, the mean spherical equivalent was +0.22 ± 0.53 D (range: −1.00 to +0.75 D) for the right eye and +0.28 ± 0.54 D (range: −0.75 to +1.25 D) for the left eye. Six months after surgery, the obtained results of MRSE did not differ significantly in comparison to 3-month results (Table 4).

### 3.4. Defocus Curve (6 Months Postoperatively)

**Figure 1:**
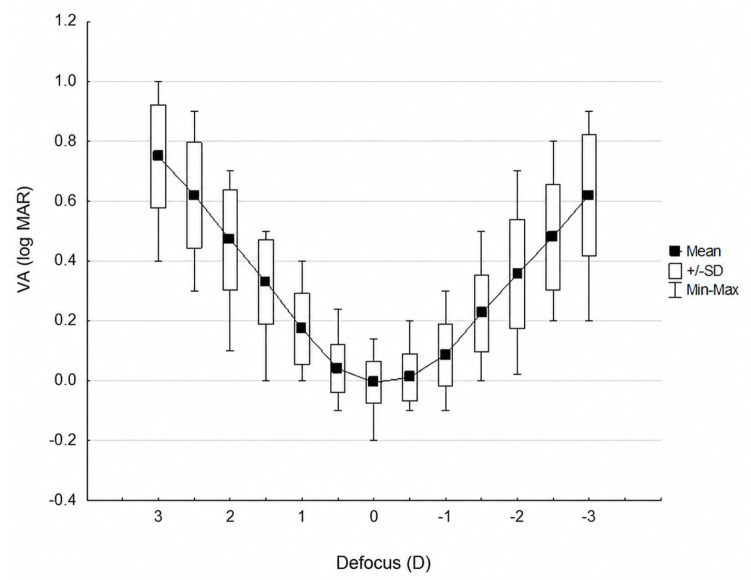
Binocular defocus curve 6 months after surgery (mean ± SD); VA—visual acuity.

The binocular defocus curve demonstrated prolonged depth of focus for intermediate distances (from +0.5 to −2.0 D) (Figure 1).

### 3.5. Contrast Sensitivity Outcomes

**Figure 2:**
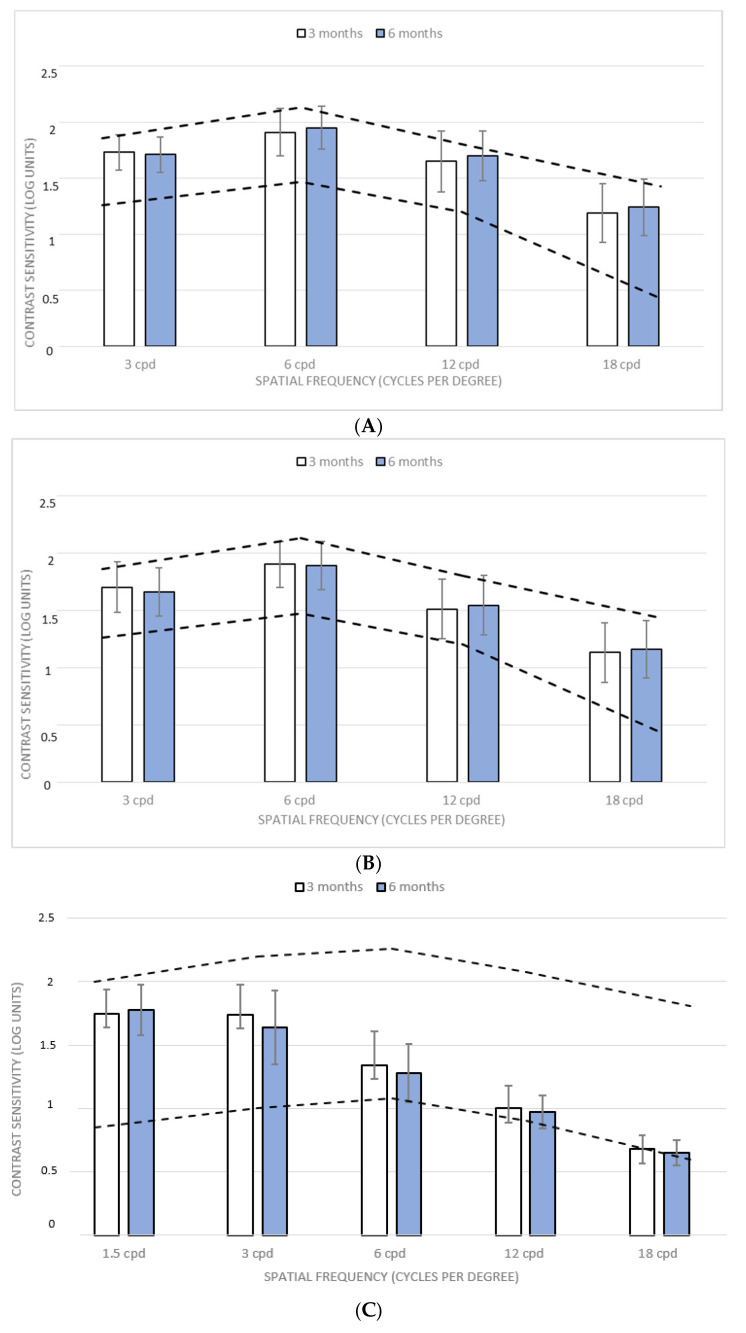
(**A**). Binocular photopic contrast sensitivity for distance (mean ± SD) 3 and 6 months after surgery. Comparison with normal range for the age group 50–75 years [9]—interrupted black line. (**B**). Binocular mesopic contrast sensitivity for distance (mean ± SD) 3 and 6 months after surgery. Comparison with normal range for the age group 50–75 years [9]—interrupted black line. (**C**). Binocular photopic contrast sensitivity for near distance (mean ± SD) 3 and 6 months after surgery. Comparison with normal range for the age-matched group 50–75 years [10]—interrupted black line.

No significant changes in contrast sensitivity for distance and near vision between the 3- and 6-month follow-up visits were found (*p* ≥ 0.08). The graphical comparison of contrast sensitivity is shown in Figure 2A–C. Detailed data are presented in Table A1, Table A2 and Table A3.

### 3.6. Visual Satisfaction Outcomes

**Figure 3:**
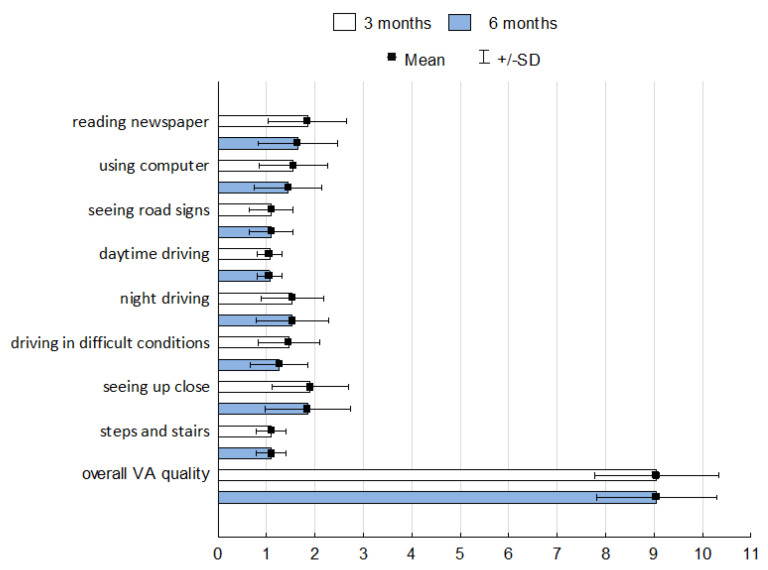
Comparison of the results of patient visual satisfaction 3 and 6 months after surgery.

Visual satisfaction was assessed using a modified Visual Function Questionnaire (VFQ-25). Overall, visual quality at six months postoperatively was high, with a mean score of 9.05. The worst results were achieved for seeing up close and reading newspapers, with 1.8 and 1.66, respectively. A comparison of visual satisfaction outcomes at 3 and 6 months is presented in Table 5 and Figure 3. No significant differences between the results of VFQ-25 test 3 and 6 months after the surgery were found (*p* ≥ 0.40).

### 3.7. Photic Phenomena

Three months postoperatively, six patients reported low-intensity glare, which did not change significantly during observation time. Similarly, in six patients, frequency and severity of halo were also low at 3 and 6 months and not statistically different (Table 6).

### 3.8. Spectacle Independence

At three and six months after surgery, all patients (100%) were independent for distance vision, 75% were independent for intermediate vision, and 45% of patients were satisfied with vision for near distance. However, all patients required corrective spectacles for optimal near vision (logMAR 0.05), with a mean addition of +2.25 ± 0.65 D.

## 4. Discussion

In this study, the obtained visual outcomes are in agreement with other study results [11] (Table A4) and suggest that during the follow-up period (range: 3–12 months), the monofocal-plus Isopure 1.2.3 IOL provides very good uncorrected binocular distance visual acuity, good intermediate vision and acceptable near vision with low frequency and severity of photic phenomena. The visual function results are also comparable for other monofocal IOLs, such as Tecnis Eyhance [5], RayOne EMV [12] or Acrysof IQ Vivity [13].

The postoperative examination 6 months after bilateral Isopure 1.2.3 IOL implantation also showed satisfactory VA results for distance, intermediate and good functional near vision. Refractive outcomes indicated a significant improvement after surgery (Table 4) and demonstrated the effectiveness of the procedure. Near vision provided by the Isopure 1.2.3 IOL, similarly to other EDOF lenses [14,15,16,17,18], was worse than after implantation of multifocal IOLs, and corrective spectacles were necessary in all cases for this distance. However, in a high percentage of patients in our study (45%), near vision was described as good and allowed for functioning during the day without spectacles, which was in agreement with other published results [19,20].

Defocus curves measure the range of useful vision provided by the IOL through the measurement of VA at various vergence levels using trial lenses of different fixed powers. In our study, at six months after surgery (Figure 1), the binocular defocus curve showed good VA at distance and intermediate ranges, with a depth of focus of approximately 1.5 D, which was consistent with previously published data after implantation of the Isopure 1.2.3 IOL [11].

Contrast sensitivity is an important complementary test for the assessment of visual function after IOL implantation. In our study, binocular contrast sensitivity was within the normal range under photopic and mesopic conditions at all measured spatial frequencies; however, not described before, near CS for high spatial frequencies was near the lower limit of normal. It was difficult to compare the CS results with other studies because different systems of CS analysis were used [12,20,21]. In our study, the CS measurements (CSV1000) were comparable to those described by Stodulka et al. [21]. The CS results after binocular implantation of the Isopure 1.2.3 IOL were also similar to those of other monofocal-plus lenses, such as RayOne EMV [12], Tecnis Eyhance [5], Micropure Monofocal IOL [20]. In our study, at six months postoperatively, overall visual quality was high (Table 5, Figure 3). All patients reported complete spectacle independence for distance vision, the majority also for intermediate vision, and almost half of the patients stated that they were satisfied with near vision; however, the satisfaction score for near vision was the lowest.

Photic phenomena (glare, halos, starbursts) are a frequent feature in patients after implantation of multifocal IOLs and might be limitations for individuals with large pupils or for patients who work in low-light conditions, especially drivers operating vehicles at night. EDOF lenses, including non-diffractive EDOF IOLs, have demonstrated a significantly reduced incidence of photic phenomena compared to multifocal IOLs and are comparable to monofocal IOLs [22,23,24,25]. In our study, six months postoperatively, the incidence of glare was observed in 25% of patients and halo was reported in 20% of cases with low severity (around 0.5). These results are comparable with those of Ang Ret et al. [23] regarding the frequency of photic phenomena. In other studies, the results were even better [14,26,27]. It is difficult to estimate the exact incidence and severity of glare and halo because other studies used non-standardized questionnaires. However, the frequency of photic phenomena after Isopure 1.2.3 and other monofocal-plus IOLs was significantly lower in comparison with multifocal IOLs (glare: 37–50%, halos: 57–85%) [22].

This lens is characterized by stabilization of visual outcomes after 3 months (Table 2, Table 3, Table 4, Table 5 and Table 6, Figure 2A–C and Figure 3) compared to multifocal IOLs (6 months and longer), which is an additional advantage of this type of IOLs. The lingual gyrus, an important component of the visual attention network, has been proposed as a circuit breaker to reorient attention to a new external information [28]. Additionally, it has a critical function in spatial memory and visual attention [29,30]. The visual input changes after implantation of monofocal-plus IOLs create a continuous range of vision. It is sensible to expect that these changes contribute to processing new types of sensory information faster than after multifocal IOL implantation.

The limitations of our study include the limited sample size of patients and the lack of standardized computer systems like Dr Kramer Halo & Glare Simulator, which for precise, repeatable, qualitative and quantitative assessment of photic phenomena (Glare, Halo) after IOL implantation. Another weakness of our study is the absence of a 3-month defocus curve; so, comparison of the results between 3 and 6 months was impossible.

## 5. Conclusions

Our results are in agreement with published data and suggest that bilateral implantation of Isopure 1.2.3 IOLs provides very good distance vision, good intermediate vision and acceptable vision for near distance. A low incidence and low perception of photic phenomena, as well as significant spectacle independence, were achieved. The absence of significant differences between follow-up points suggests complete stabilization of the investigated visual outcomes starting from the third postoperative month. This may be the result of good neuroadaptation to new optical conditions over a short period of time; however, further research in this field is needed for definitive conclusions. This lens is a very good option for patients who desire spectacle independence but cannot tolerate photic phenomena frequently associated with multifocal IOLs.

## Figures and Tables

**Table 1:** Demographic and biometric data before surgery.

Patients (*n* = 20)	
Mean age, years (range)	66.7 (51–75)
Sex, *n* (%)	
women	14 (70)
men	6 (30)
ACD (mm), mean + SD	2.99 ± 0.40
AL (mm), mean + SD	23.28 ± 0.89
K1 (D), mean + SD	43.31 ± 1.20
K2 (D), mean + SD	43.85 ± 1.23
CA (D), mean + SD	−0.53 ± 0.23
IOL power (D), mean + SD	22.63 ± 2.25
IOP (mmHg), mean + SD	16.54 ± 2.08

ACD—anterior chamber diameter; AL—Axial length; K1, K2- keratometry (D); CA—corneal astigmatism (D); D = diopters; SD—standard deviation; IOP—intraocular pressure.

**Table 2:** Comparison of uncorrected binocular visual acuity values (20 patients) and monocular visual acuity values of right and left eyes analyzed separately (20 eyes each) at 3 and 6 months after surgery for distance (UDVA), intermediate (UIVA), and near (UNVA) vision in logMAR.

	3 Months	6 Months	*p* Value
UDVA mean ± SD			
Binocular	0.02 ± 0.07	0.01 ± 0.07	0.76
Right eye	0.05 ± 0.08	0.05 ± 0.10	0.81
Left eye	0.06 ± 0.07	0.08 ± 0.09	0.61
UIVA 66 cm mean ± SD			
Binocular	0.15 ± 0.12	0.18 ± 0.12	0.35
Right eye	0.25 ± 0.16	0.26 ± 0.19	0.59
Left eye	0.21 ± 0.15	0.27 ± 0.17	0.06
UIVA 80 cm mean ± SD			
Binocular	0.19 ± 0.11	0.21 ± 0.11	0.64
Right eye	0.24 ± 0.17	0.28 ± 0.16	1.0
Left eye	0.24 ± 0.14	0.30 ± 0.17	0.06
UNVA mean ± SD			
Binocular	0.32 ± 0.12	0.35 ± 0.16	0.15
Right eye	0.46 ± 0.15	0.49 ± 0.19	0.20
Left eye	0.44 ± 0.11	0.48 ± 0.16	0.12

SD—standard deviation; UDVA—uncorrected distant visual acuity; UIVA—uncorrected intermediate visual acuity; UNVA—uncorrected near visual acuity.

**Table 3:** Comparison of binocular best-corrected visual acuity values (20 patients) and monocular best-corrected visual acuity values of right and left eyes analyzed separately (20 eyes each) at 3 and 6 months after surgery for far, intermediate and near distance in logMAR scale.

	3 Months	6 Months	*p* Value
DBCVA mean ± SD			
Binocular	−0.02 ± 0.06	−0.02 ± 0.07	0.59
Right eye	0.01 ± 0.05	0.00 ± 0.07	0.62
Left eye	0.03 ± 0.06	0.02 ± 0.07	0.66
BCIVA 66 cm mean ± SD			
Binocular	0.04 ± 0.05	0.03 ± 0.06	0.72
Right eye	0.06 ± 0.08	0.06 ± 0.09	0.97
Left eye	0.06 ± 0.06	0.09 ± 0.09	0.11
BCIVA 80 cm mean ± SD			
Binocular	0.09 ± 0.11	0.08 ± 0.08	0.64
Right eye	0.13 ± 0.12	0.13 ± 0.11	1.00
Left eye	0.14 ± 0.09	0.17 ± 0.10	0.06
BCNVA mean ± SD			
Binocular	0.07 ± 0.07	0.05 ± 0.06	0.35
Right eye	0.11 ± 0.09	0.07 ± 0.07	0.06
Left eye	0.11 ± 0.09	0.09 ± 0.06	0.72

SD—standard deviation; DBCVA—distance best-corrected visual acuity; BCIVA—best-corrected intermediate visual acuity; BCNVA—best-corrected near visual acuity.

**Table 4:** MRSE before, and 3 and 6 months after surgery.

MRSE							*p* Value *
	Pre-Op		3 Months		6 Months		
	Mean ± SD	Range	Mean ± SD	Range	Mean ± SD	Range	
OU	−0.57 ± 2.54	−5.25 to +3.25	0.26 ± 0.54	−1.00 to +1.25	0.29 ± 0.55	−1.00 to +1.25	0.70
OD	−0.91 ± 2.64	−5.25 to +3.25	0.22 ± 0.53	−1.00 to +0.75	0.32 ± 0.53	−1.00 to +0.75	0.51
OS	−0.24 ± 2.32	−4.25 to +2.75	0.29 ± 0.55	−0.75 to +1.25	0.26 ± 0.58	−1.00 to +1.25	0.88

* *p* value between mean MRSE values 3 and 6 months postoperatively.

**Table 5:** Modified VFQ-25 test results 3 and 6 months after surgery (from 1 to 5 = best to worst, VA quality 1–10 worst–best).

Parameter	3 Months	6 Months	*p* Value
	Mean ± SD	Mean ± SD	
reading newspaper	1.85 ± 0.81	1.65 ± 0.81	0.40
using computer	1.56 ± 0.70	1.44 ± 0.70	0.62
seeing road signs	1.10 ± 0.45	1.10 ± 0.45	0.99
daytime driving	1.07 ± 0.26	1.07 ± 0.26	0.98
night driving	1.53 ± 0.64	1.53 ± 0.74	0.90
driving in difficult conditions	1.47 ± 0.64	1.27 ± 0.59	0.40
seeing up close	1.90 ± 0.79	1.85 ± 0.88	0.77
steps and stairs	1.10 ± 0.31	1.10 ± 0.31	0.99
overall VA quality	9.05 ± 1.28	9.05 ± 1.23	0.96

**Table 6:** Incidence and severity of photic phenomena—the scale of 0–4, from lowest to highest severity, respectively, by modified VFQ-25 (mean ± SD).

	3 Months	6 Months	*p* Value
glare			
% of patients	30% (6/20)	25% (5/20)	0.11
severity	0.70 ± 0.98	0.50 ± 0.95	0.11
halo			
% of patients	30% (6/20)	20% (4/20)	0.36
severity	0.60 ± 1.10	0.45 ± 1.05	0.27

## Data Availability

The original contributions presented in this study are included in this article. Further inquiries can be directed to the corresponding author.

## Appendix A

**Table A1:** Contrast sensitivity for near distance (mean ± SD).

Contrast Sensitivity for Near Vision (Mean ± SD)			*p* Value
	3 Months	6 Months	
1.5 cpd	1.75 ± 0.19	1.78 ± 0.20	0.14
3 cpd	1.74 ± 0.24	1.64 ± 0.29	0.08
6 cpd	1.34 ± 0.27	1.28 ± 0.23	0.25
12 cpd	1.00 ± 0.18	0.97 ± 0.13	0.59
18 cpd	0.68 ± 0.11	0.65 ± 0.10	0.20

**Table A2:** Contrast sensitivity for far vision in photopic conditions (mean ± SD).

Contrast Sensitivity for Far Vision in Photopic Conditions (Mean ± SD)			*p* Value
	3 Months	6 Months	
3 cpd	1.73 ± 0.16	1.71 ± 0.16	0.68
6 cpd	1.91 ± 0.21	1.95 ± 0.19	0.67
12 cpd	1.65 ± 0.27	1.70 ± 0.22	0.28
18 cpd	1.19 ± 0.26	1.24 ± 0.25	0.23

**Table A3:** Contrast sensitivity for far vision in mesopic conditions (mean ± SD).

Contrast Sensitivity for Far Vision in Mesopic Conditions (Mean ± SD)			*p* Value
	3 Months	6 Months	
3 cpd	1.70 ± 0.22	1.66 ± 0.21	0.23
6 cpd	1.90 ± 0.20	1.89 ± 0.21	1.00
12 cpd	1.51 ± 0.26	1.54 ± 0.26	0.35
18 cpd	1.13 ± 0.26	1.16 ± 0.25	0.11

**Table A4:** Comparison of the reported outcomes of patients with Isopure 1.2.3., Tecnis Eyhance IC 800, Rayone EMV and Acrysof IQ Vivity.

Author (Year)	Eyes (Patients)	Time of Follow-Up (Months)	Binocular UDVA	Binocular CDVA	Binocular Intermediate 66 cm		Binocular Intermediate 80 cm		Binocular UNVA	Binocular DCNVA	Glare % of Patients (Severity)	Halo % of Patients (Severity)
					UIVA	DCIVA	UIVA	DCIVA				
Stodulka and Slovak (2021) [21]	36 (18)	6	−0.02 ± 0.13	−0.09 ± 0.06	0.20 ± 0.14	0.20 ± 0.11	0.12 ± 0.11	0.14 ± 0.08	-	-	-	-
Bova and Vita (2022) [31]	42 (21)	12	0.03 ± 0.04	0.01 ± 0.03	0.22 ± 0.06	0.21 ± 0.07	-	-	-	-	-	-
Bernabeu-Arias et al. (2023) [32]	183 (109)	4	0.02 ± 0.08	−0.02 ± 0.06	-	0.13 ± 0.11	-	0.04 ± 0.10	-	0.34 ± 0.14	-	-
Tomagova et al. (2023) [24]	124 (62)	1–2	−0.02 ± 0.07	-	-	-	0.13 ± 0.11	-	0.40 ± 0.20	-	16% +16% starburst	5%
Lesieur and Dupreye (2023) [27]	22 (22)	3	0.06 ± 0.11	0.01 ± 0.02	-	-	-	-	0.42 ± 0.18	0.40 ± 0.17	0	0
Mencucci et al. (2023) [5]	12 (12)	3	0.03 ± 0.04	0.02 ± 0.04	0.16±0.10	0.13 ± 0.08 BCIVA 0.03 ± 0.04	-	-	0.35 ± 0.07	0.32 ± 0.09 BCNVA 0.06 ± 0.05	0	0
Ang, Stodulka, Poyales (2023) [23]	130 (65)	4–6	0.00 ± 0.07	−0.05 ± 0.07	0.10 ± 0.12	0.11 ± 0.11	0.06 ± 0.11	0.07 ± 0.09	-	0.32 ± 0.12	10.34 to 18.76 *	18.31 to 27.32 *
Veselý et al. (2025) [12]	20 (10)	3	−0.07 ± 0.05	−0.08 ± 0.04	-	0.18 ± 0.09	-	0.01 ± 0.11	-	0.37 ± 0.07	-	-
Lubiński et al. (2026)	40 (20)	6	0.01 ± 0.07		0.18 ± 0.12		0.21 ± 0.11		0.35 ± 0.16		25% (0.50 ± 0.95) **	20% (0.45 ± 1.05) **
Tecnis Eyhance ICB00												
Mencucci et al. (2023) [5]	24 (12)	3	0.03 ± 0.04	-	0.14 ± 0.07	-	-	-	0.34 ± 0.04	-	0	0
RayOne EMV												
Veselý et al. (2025) [12]	18 (9)	3	−0.04 ± 0.05	-	-	0.16 + 0.10	-	0.10 ± 0.07	-	0.36 ± 0.12	-	-
AcrySof IQ Vivity												
Giannuzzi et al. (2024) [13]	80 (40)	6	0.02 ± 0.03	−0.05 ± 0.07	0.04 ± 0.07 (60 cm)	CIVA −0.03 ± 0.06 (60 cm)	−0.03 ± 0.06	CIVA −0.03 ± 0.06	0.11 ± 0.04	CNVA −0.04 ± 0.06	2.5% +blurred vision 7% + double image 7.5%	7.5%

- no data collected; 0.—no spontaneous complaints of dysphotopsia; * data from simulation software—uncomparable data; ** intensity/severity measured by VFQ-15 in the scale of 0–4, from lowest to highest subjective intensity, respectively.
